# Role of supplementary motor area in cervical dystonia and sensory tricks

**DOI:** 10.1038/s41598-022-25316-w

**Published:** 2022-12-08

**Authors:** Hyun Joo Cho, Rebecca Waugh, Tianxia Wu, Pattamon Panyakaew, Karin Mente, Demelio Urbano, Mark Hallett, Silvina G. Horovitz

**Affiliations:** 1 Human Motor Control Section, National Institute of Neurological Disorders and Stroke, National Institutes of Health, Bethesda, MD USA; 2 Department of Psychology, University of Virginia, Charlottesville, VA USA; 3 Clinical Trial Unit, National Institute of Neurological Disorders and Stroke, National Institutes of Health, Bethesda, MD USA; 4 Department of Medicine, Chulalongkorn Centre of Excellence for Parkinson’s Disease and Related Disorders, Faculty of Medicine, Chulalongkorn University and King, Chulalongkorn Memorial Hospital, Thai Red Cross Society, Bangkok, Thailand; 5 Departments of Neurology and Pathology, Case Western Reserve University, Cleveland, USA; 6 Neurology Service, VA Northeast Ohio Healthcare System, Cleveland, OH USA; 7 Cleveland Alzheimer’s Disease Research Center, Cleveland, OH USA; 8 David Geffen School of Medicine, University of California Los Angeles, Los Angeles, CA USA; 9 Charles R. Drew University of Medicine and Science, Los Angeles, CA USA

**Keywords:** Diseases of the nervous system, Motor control, Sensorimotor processing, Diseases, Neurology

## Abstract

Sensory trick is a characteristic feature of cervical dystonia (CD), where a light touch on the area adjacent to the dystonia temporarily improves symptoms. Clinical benefit from sensory tricks can be observed before tactile contact is made or even by imagination. The supplementary motor area (SMA) may dynamically interact with the sensorimotor network and other brain regions during sensory tricks in patients with CD. In this study, we examined the functional connectivity of the SMA at rest and during sensory trick performance and imagination in CD patients compared to healthy controls using functional magnetic resonance imaging. The functional connectivity between the SMA and left intraparietal sulcus (IPS) region was lower in CD patients at rest and it increased with sensory trick imagination and performance. SMA-right cerebellum connectivity also increased with sensory trick imagination in CD patients, while it decreased in healthy controls. In CD patients, SMA connectivity increased in the brain regions involved in sensorimotor integration during sensory trick performance and imagination. Our study results showed a crucial role of SMA in sensorimotor processing during sensory trick performance and imagination and suggest the IPS as a novel potential therapeutic target for brain modulation.

## Introduction

Sensory tricks are maneuvers that can temporarily relieve dystonic symptoms by applying sensory stimulation on an area adjacent to dystonia^1^. Typical stimuli are light touches to a particular skin area; this touch is often sufficient even when the force applied is weak and ordinarily could not counteract dystonic contractions simply by overpowering them. Some tricks, however, do need forceful counterpressure. Additionally, contact with the face is not always necessary – dystonia can be alleviated by hand movement toward the face before tactile stimulation occurs and sensory tricks with mental imagination can be partially effective as well^2^. For the latter reason, sensory tricks are also called alleviating maneuvers. Previous studies found that sensory tricks are effective in most cervical dystonia (CD) patients, ranging from 75 to 90%^3,4^. Despite their high prevalence, the mechanism has remained elusive.

Previous neurophysiological studies have attempted to elucidate the mechanism of sensory tricks. One transcranial magnetic stimulation (TMS) study using double-pulse stimulation on the motor cortex found that intracortical facilitation was significantly increased in CD patients compared to healthy controls (HC), and that the use of sensory tricks decreased the abnormal intracortical facilitation in CD patients^5^. Our group showed that the contingent negative variation (CNV) – a brain potential produced during motor preparation in an experimental paradigm of warning and imperative signal – was significantly increased when CD patients performed sensory tricks compared to HC who mimicked similar movements^6^. A case report using magnetoencephalography to record the sensory trick in a patient with CD showed that the brain coherence level for gamma frequency increased with sensory tricks, suggesting increased GABAergic activity^7^. Another study from our group demonstrated that the gamma band corticocortical coherence between premotor and sensorimotor areas increased during sensory trick but decreased during voluntary neck movement, while opposite trends were observed in HC^8^.

Neuroimaging approaches have been used to identify the neuroanatomical basis for sensory tricks. An H_2_^(15)^O positron emission tomography study of seven cases of CD showed that performing a sensory trick was associated with increased activity in the superior and inferior parietal lobules and reduced activity of the supplementary motor area (SMA) and primary sensorimotor cortex^9^. A recent fMRI study showed that sensory trick performance was associated with decreased sensorimotor network connectivity and that the cerebellum was activated with sensory trick imagination in patients with sensory tricks compared to patients with no sensory tricks^10^.

These studies indicate that sensory tricks utilize a cortical network involved in motor preparation and sensorimotor integration. Evidence from neurophysiology and neuroimaging studies as well as clinical observation suggest the role of the SMA in sensory tricks. SMA is responsible for the planning of movements and is known to be a main generator for the CNV^11^. However, previous studies could not reveal the role of SMA in sensory tricks in relation to whole brain connectivity. In this study, we sought to examine the functional connectivity of the SMA at rest and during sensory trick performance and imagination in CD patients compared to HC using fMRI. We hypothesized that SMA would dynamically interact with the sensorimotor network and other brain regions during sensory tricks in CD.

## Methods

### Subjects

Twenty-three patients with CD and sensory tricks (age range: 31–76; 12 males/11 females) were recruited from the movement disorders clinic at the National Institutes of Health (NIH) between 2012 and 2020. Twenty-three age-matched HC (age range: 32–82; 11 males/12 females) were recruited from the local community during the same period. None of the subjects had other neurological or psychiatric disorders nor history of substance abuse or traumatic brain injury. All subjects were examined by board-certified neurologists and had a clinical MRI less than one year before this study. CD patients completed the Toronto Western Spasmodic Torticollis Rating Scale (TWSTRS). Sensory trick effectiveness was confirmed by clinical exam. Seven patients were noted to have head tremors, however none of them were considered as tremor dominant CD. Subjects provided written informed consent for this study, which was approved by the NIH Institutional Review Board (Combined NeuroScience Institutional Review Board) and NINDS ethics committee (Deputy Ethics Counselor-NINDS committee). All research was performed in accordance with the Declaration of Helsinki and relevant guidelines/regulations.

### MRI acquisition

Structural and functional MRI scans were collected on a 3-Tesla MRI scanner (Siemens Skyra, Munich, Germany) using a 32-channel receiver head coil. For all subjects we acquired a T1-weighted structural scan. For most subjects, the structural scan was a multi-echo magnetization prepared rapid gradient echo with a repetition time (TR) of 2530 ms, echo time (TE) of 1.69/3.55/5.41/7.27 ms, inversion time of 1100 ms, and flip angle of 7 degrees. In five subjects, a magnetization prepared rapid gradient echo (MPRAGE) with a single TE was collected instead, with a TR of 3000 ms, TE of 3.03 ms, inversion time of 900 ms, and flip angle of 9 degrees. In both cases, voxel size was 1 mm × 1 mm × 1 mm. In all subjects, at least one single-shot echo planar imaging scan was collected with a TR of 2000 ms, TE of 11 ms, flip angle of 70 degrees, and voxel size of 3 mm x 3 mm x 3 mm in 180-time steps for fMRI. Scanning time was approximately 6 min.

Subjects abstained from both alcohol and caffeine for at least 48 h before MRI. CD patients’ MRI scans were at least 11 weeks after their last botulinum toxin injection. All subjects participated in resting state-fMRI (rs-fMRI) during which they were instructed to lie motionless with eyes closed and without falling asleep. A subgroup of 10 patients and 11 controls completed additional task-based fMRI scans with identical scan parameters. In the first of two fMRI scans, the “touch” scan, CD patients performed their sensory trick for the entire scan duration. To maintain the effectiveness of the sensory trick, patients were allowed to adjust their finger position slightly without moving their head. Secondly, in the “imagine” scan, they actively imagined performing their sensory trick throughout the scan. HC mimicked the sensory trick of the patient to whom they were matched for the “touch” and “imagine” scans. We applied a head frame and pads between the head and the frame to minimize the head movement in the scanner.

### fMRI pre-processing

Skull-stripped T1-weighted MPRAGE images were warped to the N27 Colin brain template. The fMRI scans were preprocessed with AFNI^12^. Functional data were preprocessed with steps which included removing the first two timepoints from the scan to ensure magnetization steady state, despiking, slice time correction, and motion correction with a motion limit of 3 mm. B0 unwarping was not performed on our dataset, however, we ran quality check on raw data which revealed no significant distortion. The data were linearly aligned to the N27 Colin space using parameters derived from the structural alignment. Functional data were then spatially smoothed with a 3 mm full width at half maximum filter, and bandpassed at 0.01 < *f* < 0.1 Hz to capture the resting-state fluctuations of the blood oxygenation level-dependent signal. Based on motion parameters established prior to analysis, two HC with excessive head motion (average motion > 0.3 mm) in their rs-fMRI scans were excluded. We also excluded one patient because of technical problems that prevented further fMRI processing. Therefore, a total of 20 CD patients were included in the resulting analysis, along with 20 matched controls.

### Seed connectivity analysis

Two seeds were placed bilaterally in the SMA with Talairach X, Y, Z coordinates (in N27 Colin template) of -3, -4, 54 and 2, -4, 54 for the left and right SMA, respectively^9^. Seeds were expanded spherically with a radius of 3 mm. We extracted the mean timeseries of the voxels within each seed, then calculated the Pearson’s r correlation between the timeseries and all other voxels across the whole brain. This produced two statistical maps per subject for each functional scan (rest, imagine, and touch).

### Statistical analysis

A linear mixed effects (LME) model was constructed to analyze the statistical maps of functional connectivity. This multivariate model allows for a different number of subjects in each condition, allowing for the inclusion of both the subjects who completed just the resting state scan and those who also completed trick and imagine scans^13^. The model tested the main effects and interactions between the group (PT or HC) and task (rest, touch or imagine). Hemisphere of seed (left or right) and age were included as covariates.

The model produced a statistical value (Z-score) at each voxel within the brain. To determine significance, we first applied a whole-brain voxel-wise threshold of p < 0.001 uncorrected to the output maps for the group, task, side of the seed, and the interaction between the group and task. AFNI’s 3dClustSim tool then estimated the number of contiguous significant voxels required to exceed a family-wise error-corrected significance threshold of p < 0.05. The mean values of surviving clusters were extracted for each subject. The mean values were computed using only non-zero voxels.

Repeated measures analysis of covariance was performed to evaluate the effect of the two explanatory factors (group and task) on mean Z-scores of surviving clusters, where the covariates were age and sex, the between-subject factor was group, and the within-subject factor was task. For each outcome variable, the Bonferroni method was used to adjust for the four comparisons of interest: (1) touch versus rest, and (2) imagine versus rest for both PT and HC group. Additionally, the adjusted p-values for 60 comparisons (4 per outcome variable) were calculated using Hommel’s method to control the family error rate. Shapiro–Wilk’s test for normality was conducted based on the model residuals. The sensitivity analysis was also performed by excluding the subjects with extreme outliers, which were defined as: the absolute studentized residuals greater than 3. SAS 9.4 was used for the statistical analyses.

## Results

### Patient demographics

The mean age for CD patients was 58 years at the time of study participation (age range: 31–76; 12 males/11 females). The man age of CD onset was 42 (age range: 13–69). The patient demographics are summarized in Table 1. There were no differences in head motion parameters between groups.

**Table 1:** Demographics of cervical dystonia patients.

Patient ID	Age	Sex	Age at onset	Disease duration	TWSTRS-severity	TWSTRS-disability	TWSTRS-pain	TWSTRS-total	Head turn	Sensory trick**	
										Face touch	Hand used
1	64	M	51	13	7	1	8	16	Left torticollis	Right	Right
2*	57	M	52	5	29	8	4.25	41.25	Left laterocollis, anterocollis	Chin	Right
3	54	F	44	10	23	8	4.25	35.25	Left torticollis	Right	Right
4	76	M	66	10	15	3	10.75	28.75	Left torticollis	Left	Left
5	71	M	69	2	14	6	1.25	21.25	Right torticollis, anterocollis	Right	Right
6	31	M	13	18	23	4	10	37	Left torticollis, anterocollis	Left	Left
7	58	F	47	11	20	0	1.25	21.25	Left torticollis, laterocoolis	Left	Left
8	54	M	47	7	11	3	6.5	20.5	Right torticollis	Right	Right
9	51	F	35	16	15	3	0	18	Right torticollis	Left	Left
10	73	F	40	33	13	4	7.5	24.5	Right laterocollis	Left	Left
11	41	F	28	13	18	4	8	30	Left torticollis, anterocollis	Left	Right
12	44	M	29	15	24	12	5	41	Left torticollis	Left	Left
13	32	M	27	5	3	2	4.75	9.75	Right torticollis	Right	Right
14	71	M	62	9	6	0	0	6	Right torticollis	Right	Right
15	51	M	26	25	11	4	0	15	Left torticollis	Right	Right
16	61	F	60	1	10	5	5	20	Right torticollis	Right	Right
17	74	F	54	20	9	0	0	9	Right torticollis	Back***	Either
18	59	F	29	30	6	8	5	19	Right torticollis	Right/chin	Right
19	54	F	50	4	19	11	2.75	32.75	Right torticollis, anterocollis	Chin	Either
20	72	F	56	2	17	4	4	25	Right laterocollis, anterocollis	Chin	Either
21	58	M	18	48	17	9	3.25	29.25	Right torticollis	Right	Right
22	66	M	18	48	24	3	0	27	Right laterocollis	Left	Left
23	60	F	45	15	19	22	17	58	Right torticollis, laterocollis	Back***	Either

*Patient 2 was excluded from the analysis due to motion artifact.

**All subjects had sensory tricks but only first 11 subjects underwent additional scans for ‘touch’ and ‘imagine’.

***Back of the head and neck.

### Left versus right SMA seed

When the whole brain was compared by the side of the SMA seed, there were no clusters that passed the whole-brain voxel-wise threshold of p < 0.001. We further ran a Pearson correlation analysis between the time series using the left and right SMA; the correlation coefficient value was 0.7, suggesting strong correlation. We extracted Z-scores separately for left and right SMA seed and examined the difference statistically. There were no statistical differences in F-test for the group, task, and group x task interaction between the left and right SMA seed. When comparing between the groups and tasks, 7 out of 60 pairs of comparisons showed a difference in p value strength, but the direction of change was the same for the SMA connectivity (Supplemental Figure). Therefore, we decided to combine the values from left and right SMA seed together for further analysis.

### Functional connectivity of SMA at rest in cervical dystonia versus healthy controls

**Figure 1:**
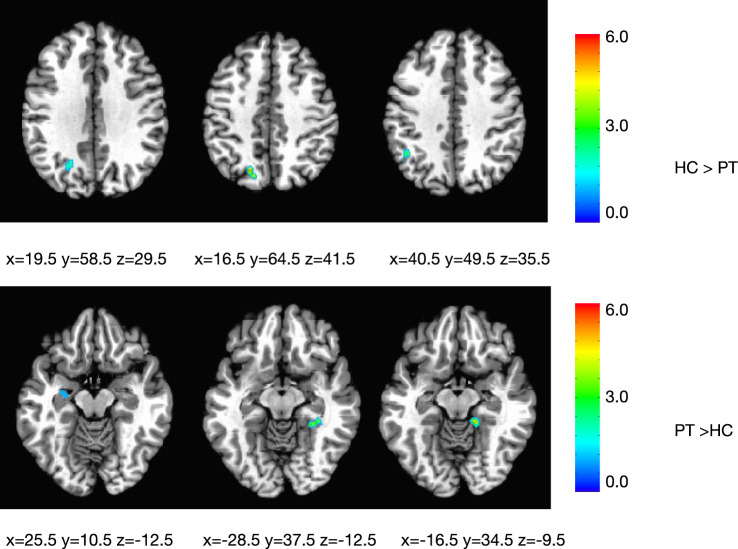
SMA connectivity at rest. (**A**) Left intraparietal sulcus and precuneus in patients were lower compared to HC. (**B**) Left hippocampus, right parahippocampal cortex and right fusiform gyrus in patients were higher compared to HC.

All 23 CD patients and age-matched HC completed rs-fMRI. Seed based analysis showed that functional connectivity between the SMA and left intraparietal sulcus (IPS)/precuneus in patients was lower compared to HC (Fig. 1A). Patients had increased connectivity between the SMA and medial temporal region (right parahippocampal cortex, right fusiform gyrus, and left hippocampus) compared to HC (Fig. 1B). The Z-scores of these regions did not correlate with disease duration or total TWSTRS score.

### Changes of SMA connectivity with sensory trick performance and imagination

**Figure 2:**
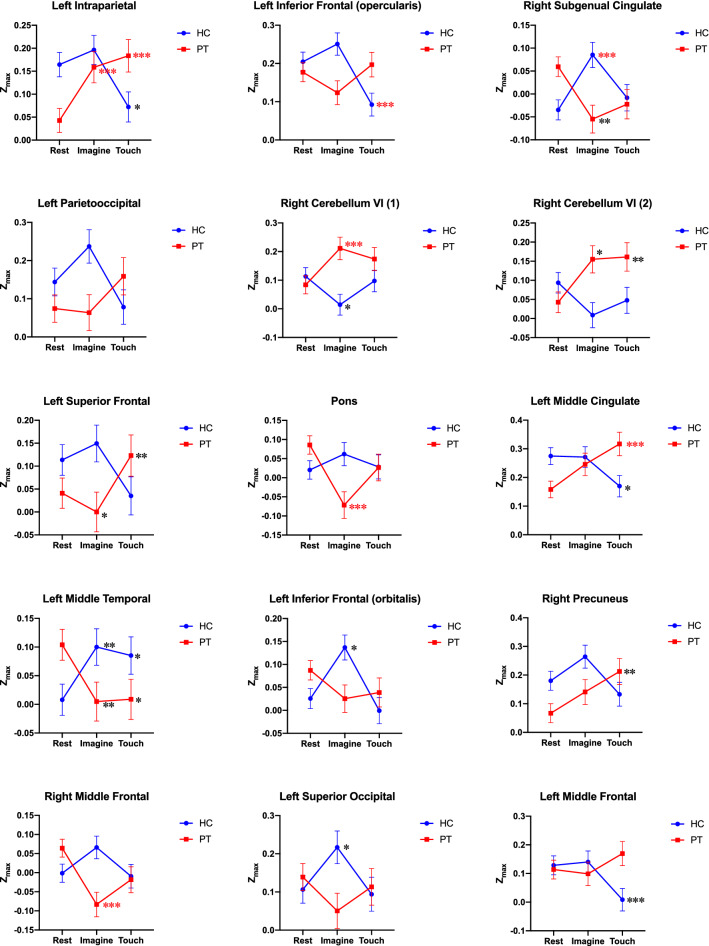
Clusters significant for group x task interaction. *p < 0.05, **p < 0.01, ***p < 0.005, ****p < 0.0001. Red asterisk indicates that statistical significance remained after Hommel’s method to control the family error rate for 60 comparisons (4 pairs for 15 clusters). Cluster location described in Table 2.

A subgroup of 11 patients and 11 controls completed the additional trick fMRI. One patient was excluded from the analysis due to motion artifact. The LME model showed 15 clusters that were significant for the group (PT vs HC) x task (rest, touch, or imagine) interaction (Fig. 2). Their locations were extracted in Talairach coordinates in N27 Colin template (Table 2).

**Table 2:** Locations of 15 clusters from Fig. 2 in Talairach coordinates in N27 Colin template.

Location	Voxels	CM x	CM y	CM z	Peak x	Peak y	Peak z
Intraparietal sulcus	55	35.3	58.8	34.7	34.5	55.5	26.5
Lt inferior frontal gyrus (opercularis)	47	42.9	− 6.5	27.6	46.5	− 7.5	17.5
Rt anterior cingulate (subgenual)	34	− 4.4	− 15.3	− 3.9	− 7.5	− 16.5	− 12.5
Lt Parietooccipital sulcus	24	6.1	60.6	14	4.5	64.5	8.5
Rt Cbll VI (1)	22	− 13.5	66.7	− 15.2	− 10.5	61.5	− 18.5
Rt Cbll VI (2)	18	− 23.3	66	− 21.8	− 22.5	64.5	− 24.5
Lt. superior frontal gyrus	16	20.2	− 60.2	10.9	19.5	− 58.5	5.5
Pons	15	− 2.9	23.5	− 31.1	− 4.5	19.5	− 33.5
Left cingulate motor area	15	3.3	40.1	44.9	1.5	37.5	41.5
Left middle temporal gyrus	14	49.1	1.5	− 17.9	46.5	− 1.5	− 21.5
Left inferior frontal gyrus (orbitalis)	14	19.9	− 19.5	− 14	13.5	− 19.5	− 18.5
Right precuneus	14	− 3.2	65.8	30.6	− 1.5	64.5	23.5
Right middle frontal gyrus	13	− 25.7	3.6	39.7	− 25.5	1.5	35.5
Left superior occipital gyrus	11	6.7	97	2.8	7.5	97.5	− 3.5
Left middle frontal gyrus	11	23.3	− 4	45.3	22.5	− 7.5	44.5

CM, center of mass; Voxels, number of voxels in each cluster.

While performing the sensory trick by touching the face, CD patients and HC showed changes in functional connectivity to the SMA in opposite directions. In patients, the SMA connectivity increased in IPS compared to rest, while it decreased in HC. Also, SMA connectivity to the left middle temporal gyrus decreased in patients with sensory trick, while it increased in HC. Moreover, SMA connectivity in the right cerebellum, left middle cingulate cortex (cingulate motor area), and right precuneus increased in patients with sensory trick, while decreasing in the left inferior frontal (ventral premotor) and cingulate motor area in HC.

Significant group x task interactions were also found for the sensory trick imagination condition. In patients, left IPS and right cerebellum had increased connectivity to SMA with imagination compared to rest. In HC, the subgenual cingulate cortex, left middle temporal, and left inferior frontal (ventral premotor) showed increased SMA connectivity. In contrast, patients were found to have decreased SMA connectivity in the pons, left middle temporal gyrus, right middle frontal gyrus (dorsal premotor), and right subgenual cingulate cortex, whereas HC showed decreased connectivity in the right cerebellum.

**Figure 3:**
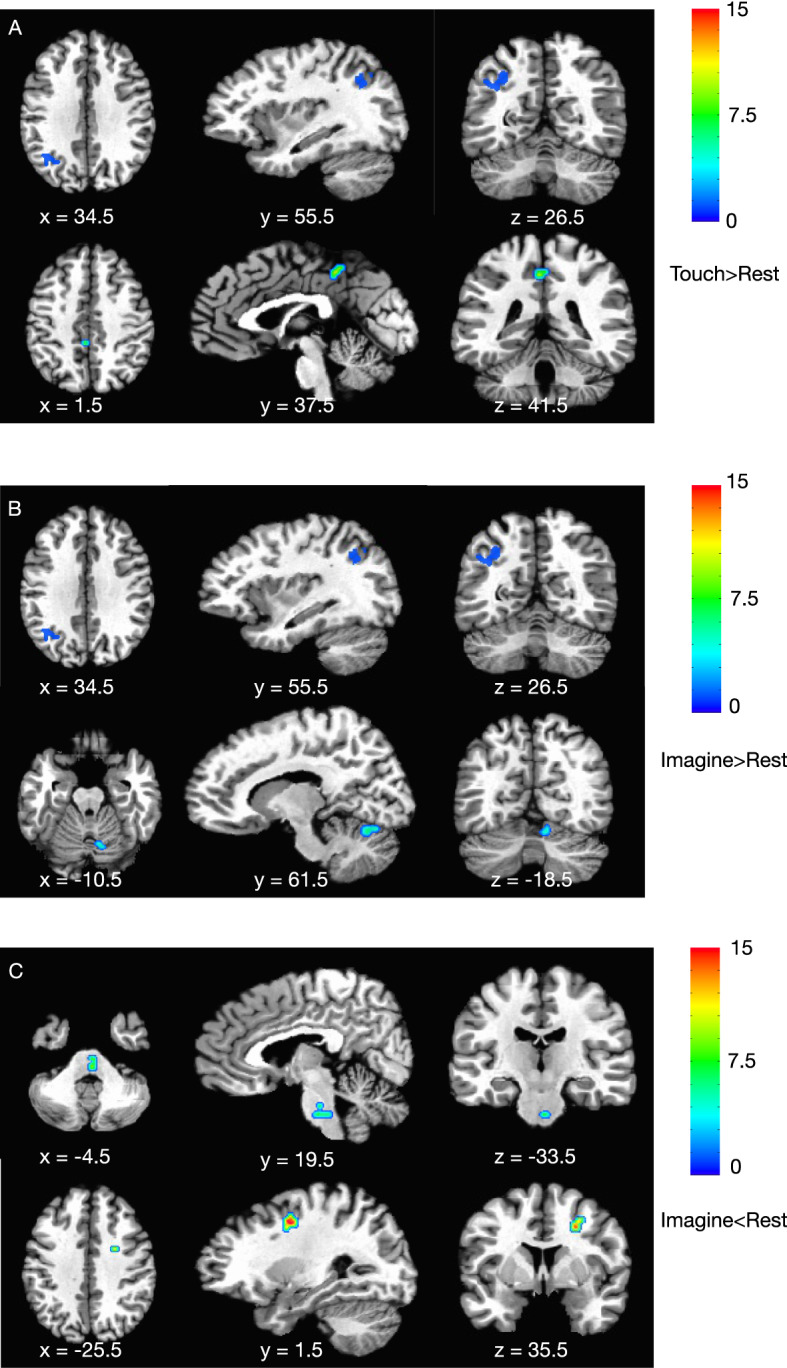
Clusters that survived Hommel’s method. (**A**) Left IPS and left middle cingulate cortex were higher with sensory trick performance in patients. (**B**) Left IPS and right cerebellum were higher with trick imagination in patients. (**C**) Pons and right middle frontal gyrus were lower with trick imagination in patients.

After adjusting for multiple comparisons of 60 pairs (HC rest vs touch, HC rest vs imagine, PT rest vs touch, PT rest vs imagine for 15 clusters each) using Hommel’s method, left IPS and left middle cingulate cortex remained significantly higher with sensory trick performance in patients with CD (Fig. 3A). For changes between the rest and imagine conditions, left IPS and right cerebellum were still significantly higher (Fig. 3B), while pons and right middle frontal gyrus were lower with trick imagination in patients (Fig. 3C).

## Discussion

Dystonia is increasingly considered a network disorder extending beyond the basal ganglia system and even the motor system^14^. Studies have shown that abnormal high-order motor processing in dystonia might occur upstream from the motor cortex. A TMS study showed that functional coupling between the posterior parietal cortex and motor cortex is abnormal in dystonic patients^15^. Somatosensory impairment in dystonia is also demonstrated by an abnormal temporal discrimination threshold^16^. fMRI studies have shown more widespread network abnormalities with pronounced reduced connectivity within the sensorimotor and frontoparietal network^14^. Abnormal high-order sensorimotor integration was again highlighted in an fMRI study that showed increased intrinsic connectivity within the left parietal lobe at rest and decreased functional connectivity between the parietal and pre/post central gyrus^17^. Clinical observation of sensory tricks in CD also suggests a role for sensorimotor integration in the pathophysiology of CD, although its mechanism has not been clearly understood.

To study the mechanism of sensory tricks in CD, our group previously performed an electroencephalogram experiment to record brain potentials during motor preparation and execution of sensory tricks using a CNV paradigm of warning and imperative signal. The late CNV was significantly larger during motor preparation of sensory tricks than during voluntary neck movements. This demonstrated that anticipatory activities in the premotor/motor area are modulated before executing the trick^6^. When we examined different cortical areas including motor (C3 or C4), somatosensory (P3 or P4), and SMA/premotor (FCz, FC5, or FC6) before and during sensory tricks, the corticocortical coherence increased between premotor and motor/sensory regions during the late preparatory phase and it increased between sensory and premotor/motor regions during the execution phase when compared to that of voluntary neck movement^8^.

The SMA is associated with motor preparation as well as execution or suppression of intended movements^18,19^. The SMA has been implicated in the pathophysiology of dystonia, evidenced by fMRI studies that showed reduced SMA connectivity in patients with dystonia^14,20^, as well as case reports of acquired dystonia due to SMA lesion in patients who had existing remote lesions in the putamen^21,22^. Our current fMRI study results showed direct evidence that SMA plays a crucial role during sensory tricks in CD. Our results showed that SMA connectivity with brain regions of sensory integration was different at rest between HC and patients. Furthermore, the SMA modulated the process of sensorimotor integration during sensory trick performance and imagination. Sensory trick effect may involve high order sensorimotor processing that are ultimately connected to GPi^23^, and SMA may play a critical role in sensory tricks by engaging with known dystonia network^24^.

The most compelling finding from our study is how connectivity changes between the SMA and posterior parietal cortex during sensory tricks. CD patients had decreased connectivity between the SMA and IPS region and precuneus at rest, but the connectivity increased with sensory trick performance and imagination. Which of the two factors, the dystonia or the presence of a sensory trick, is associated with altered SMA connectivity cannot be deduced from these results. Patients with dystonia may have an increased motor effort and thus altered afferent feedback is to be expected in dystonia. This may explain the finding of different resting state connectivity in the sensorimotor system. Given the clinical benefit associated with sensory trick, this change suggests the possibility of normalization of a dysfunctional ground state of SMA-posterior parietal cortex connectivity. The posterior parietal cortex integrates multimodal sensory information to localize the body and external objects in space, and its output goes to the motor system to execute planned movements^25^. The IPS divides posterior parietal cortex into dorsal superior parietal lobule and ventral inferior parietal lobule, and it is involved in selection between competing stimuli^26,27^. The precuneus also serves an important function in the process of sensorimotor integration^28^. Both the IPS and precuneus are activated in multimodal sensorimotor tasks^29^. Our result indicates that SMA may play a key role in sensory tricks in CD by partially correcting aberrant sensorimotor processing.

A similar trend was found for the right cerebellar lobule VI– SMA connectivity with sensory trick performance and imagination. It increased for both sensory trick performance and imagination in CD patients, although its change with trick performance did not survive the statistical correction for multiple comparisons. The difference between SMA-cerebellar connectivity in CD patients with sensory tricks compared to HC adds to the growing evidence supporting cerebellar involvement in the pathophysiology of dystonia as an active component of sensorimotor integration^30^. Additionally, fMRI studies have shown independent prefronto-cerebellar circuits in human brain^31,32^. Our study indicated that the prefronto-cerebellar circuit might work differently in CD patients compared to HC in response to motor, imaginary, and light touch. The middle cerebellar peduncle receives afferent input via pons from prefrontal and parietal pathways as well as anterior cingulate^33^. It is important to note that patients showed decreased SMA-pons connectivity with trick imagination but showed increased SMA-cerebellum connectivity, which may suggest different corticopontocerebellar pathways are engaged for motor performance and imagination.

Another notable result from our rs-fMRI was increased connectivity between the SMA and hippocampus/parahippocampal region in CD patients compared to HC. The medial temporal lobe is associated with encoding spatial- and self- motion relative to environment to monitor inertial navigation^34^. CD patients may exert extra effort for inertial navigation at rest due to abnormal head and neck position. However, the significance of this finding is unclear considering the lack of any correlation with disease duration or severity.

Overall, our results clearly showed the differences in SMA connectivity in HC and CD patients during sensory trick performance and imagination. HC had higher connectivity with the SMA in the regions of visual processing (left occipital and middle temporal) with sensory trick imagination, whereas patients showed more changes in areas of sensorimotor integration (left IPS and right cerebellum) for both tasks. Our results align with a previous study that explained sensory tricks as the presence of a cortical adaptive mechanism involving the posterior parietal cortex evidenced by better performance in temporal sensory discrimination in CD patients with effective sensory tricks compared to patients without sensory tricks^35^. Our study adds evidence that the SMA and cerebellum also play an important role in effective sensorimotor integration as a cortical adaptive mechanism.

Our study has a few limitations. We did not measure the objective degree of clinical benefit from sensory tricks (ie. EMG activity and/or gyroscope) apart from one questionnaire on TWSTRS. Therefore, we could not draw conclusions as to whether SMA connectivity change was correlated with the effectiveness of the sensory trick. The small number of patients made the clinical correlation difficult as well. Finally, our study did not include patients without sensory trick.

Our study has potential implications for a therapeutic intervention. One could consider augmenting or enhancing functional connectivity between the SMA and posterior parietal cortex to mimic the changes associated with an effective sensory trick. The IPS is a hub to process tactile stimulation for motor control^27^. The IPS could be a potential novel target for neuromodulation for CD, and the temporal discrimination threshold could be measured as a behavioral correlate.

## Supplementary Information

Supplementary Information.

## Data Availability

The data that support the findings of this study are available from the corresponding author upon reasonable request.
