# Supplementary material for: Role of supplementary motor area in cervical dystonia and sensory tricks
Source: Sci Rep. 2022 Dec 8;12:21206. doi: 10.1038/s41598-022-25316-w (PMC9731945; doi:10.1038/s41598-022-25316-w)
Supplement: Supplementary file 1 — Supplementary Information. [file 41598_2022_25316_MOESM1_ESM.docx]

0.3

0.2

**Zmax**

0.1

# Left Intraparietal


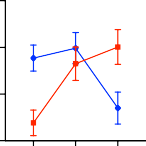

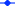

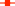
HC PT

✽✽✽✽

✽✽✽

# Left Inferior Frontal (opercularis)


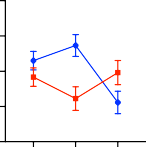
0.4

##
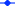
HC


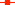
**0.3** PT

0.2

**Zmax**

# Right Subgenual Cingulate

0.15


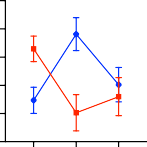


✽✽✽

✽✽

##
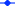
HC


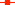
**0.10** PT

0.05

**Zmax**

0.00

0.25

0.20

0.15

**Zmax**

0.10

# Left Intraparietal

##
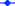

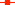
HC PT


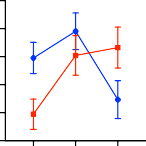


✽✽✽

✽✽

# Left Inferior Frontal (opercularis)

0.3


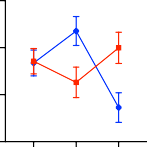


✽

✽✽✽

##
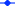
HC


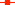
PT

0.2

**Zmax**

0.1

# Right Subgenual Cingulate

0.15


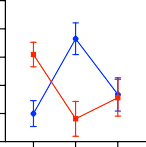


✽✽✽✽

✽✽

##
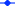
HC


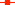
**0.10** PT

0.05


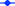


**Zmax**

0.00

✽✽ **0.1**

✽✽✽✽

-0.05

0.05

-0.05

0.0

Rest Imagine Touch

0.0

Rest Imagine Touch

-0.10

Rest Imagine Touch

0.00

Rest Imagine Touch

0.0

Rest Imagine Touch

-0.10

Rest Imagine Touch

0.3

0.2

**Zmax**

0.1

0.0

# Left Parietooccipital

##
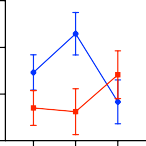

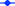

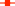
HC PT

Rest Imagine Touch

0.3

0.2

0.1

**Zmax**

0.0

-0.1

# Right Cerebellum VI (1)


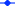

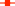
HC PT


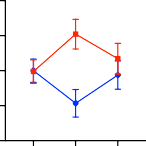


✽

Rest Imagine Touch

0.20

0.15

0.10

**Zmax**

0.05

0.00

-0.05

# Right Cerebellum VI (2)

##
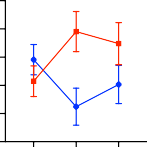

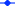

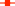
HC PT

Rest Imagine Touch

0.4

0.3

0.2

**Zmax**

0.1

0.0

# Left Parietooccipital

##
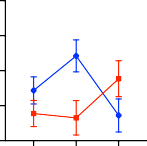

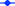

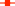
HC PT

Rest Imagine Touch

0.3

0.2

0.1

**Zmax**

0.0

-0.1

# Right Cerebellum VI (1)


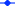

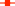
HC PT


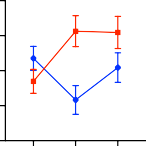


✽✽

✽

✽

Rest Imagine Touch

0.3

0.2

0.1

**Zmax**

0.0

-0.1

# Right Cerebellum VI (2)

##
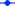

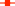
HC PT


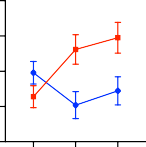


✽

✽✽✽

Rest Imagine Touch

0.20

0.15

0.10

**Zmax**

0.05

0.00

-0.05

# Left Superior Frontal

##
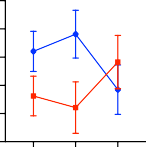

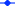

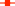
HC PT

Rest Imagine Touch

0.15

0.10

0.05

**Zmax**

0.00

-0.05

-0.10

-0.15

# Pons

##
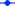

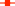
HC PT


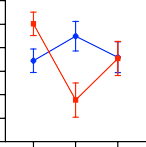


✽✽✽✽

Rest Imagine Touch

0.4

0.3

0.2

**Zmax**

0.1

0.0

# Left Middle Cingulate

##
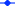

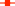
HC PT


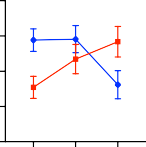


✽

✽✽

Rest Imagine Touch

0.3

0.2

0.1

**Zmax**

0.0

-0.1

# Left Superior Frontal

##
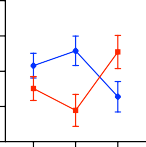

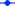

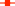
HC PT

Rest Imagine Touch

0.15

0.10

0.05

**Zmax**

0.00

-0.05

-0.10

-0.15

# Pons

##
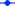

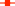
HC PT


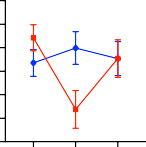


✽✽✽

Rest Imagine Touch

0.5

0.4

0.3

**Zmax**

0.2

0.1

0.0

# Left Middle Cingulate

##
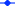

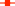
HC PT


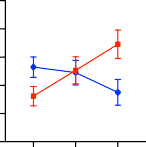


✽✽✽

Rest Imagine Touch

0.20

0.15

0.10

**Zmax**

0.05

0.00

-0.05

# Left Middle Temporal

##
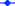

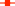
HC PT


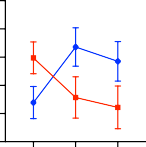


✽

Rest Imagine Touch

# Left Inferior Frontal (orbitalis)

0.20


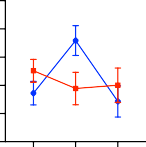


✽

##
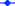
HC


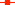
**0.15** PT

0.10

**Zmax**

0.05

0.00

-0.05

Rest Imagine Touch

0.4

0.3

0.2

**Zmax**

0.1

0.0

# Right Precuneus

##
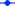

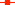
HC PT


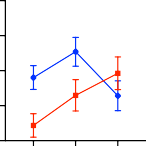


✽✽

Rest Imagine Touch

0.15

0.10

0.05

**Zmax**

0.00

-0.05

-0.10

# Left Middle Temporal

##
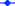

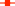
HC PT


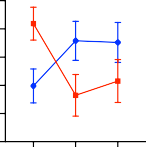


✽

✽✽✽

Rest Imagine Touch

# Left Inferior Frontal (orbitalis)

0.20


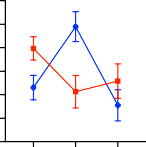


✽

##
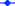
HC


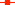
**0.15** PT

0.10

**Zmax**

0.05

0.00

-0.05

-0.10

Rest Imagine Touch

0.4

0.3

0.2


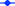


**Zmax**

0.1

0.0

# Right Precuneus

##
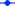

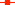
HC PT


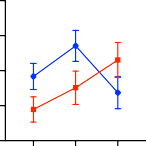


✽

Rest Imagine Touch

0.15

0.10

0.05

**Zmax**

0.00

-0.05

-0.10

-0.15

# Right Middle Frontal

##
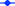

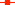
HC PT


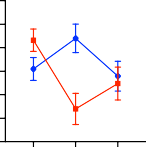


✽✽✽✽

Rest Imagine Touch

0.3

0.2

**Zmax**

0.1

0.0

# Left Superior Occipital

##
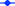

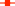
HC PT


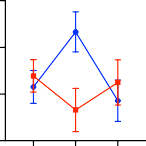


✽

Rest Imagine Touch

0.3

0.2

0.1

**Zmax**

0.0

-0.1

# Left Middle Frontal

##
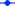

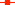
HC PT


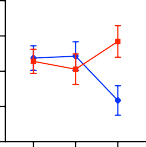


✽✽

Rest Imagine Touch

0.10

0.05

0.00

**Zmax**

-0.05

-0.10

-0.15

# Right Middle Frontal

##
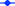

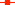
HC PT


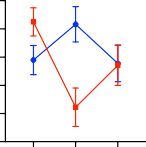


✽✽✽✽

Rest Imagine Touch

0.3

0.2

0.1

**Zmax**

0.0

-0.1

# Left Superior Occipital

##
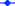

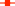
HC PT


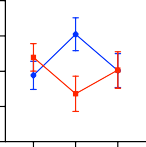


✽

Rest Imagine Touch

0.3

0.2

0.1

**Zmax**

0.0

-0.1

# Left Middle Frontal

##
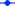

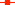
HC PT


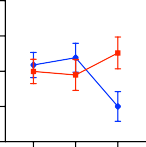


✽

Rest Imagine Touch

1. Left SMA (B) Right SMA

Supplemental Figure. Clusters significant for group x task interaction for functional connectivity using (A)left SMA and (B)right SMA seed. **p*<0.05, ***p*<0.01, ****p*<0.005, *****p*<0.0001. Red asterisk indicates that statistical significance remained after Hommel’s method to control the family error rate for 60 comparisons (4 pairs for 15 clusters). Cluster location described in Table 2 in the main text.
